# Characteristics and Outcomes of Clinical Trials on Gene Therapy in Noncongenital Cardiovascular Diseases: Cross-sectional Study of Three Clinical Trial Registries

**DOI:** 10.2196/33893

**Published:** 2022-04-21

**Authors:** Witold Pinczak, Sylwia Trzcińska, Mikołaj Kamiński

**Affiliations:** 1 Department of Medicine I Poznan University of Medical Sciences Poznań Poland; 2 Provincial Hospital in Poznan Poznań Poland

**Keywords:** gene therapy, cardiovascular disease, clinical trials, trial design, heart disease, clinical trial, therapy, cardiac risk factor, health intervention

## Abstract

**Background:**

Cardiovascular diseases remain the leading cause of morbidity and mortality worldwide. Gene therapies (GTs) may become a novel therapeutic option for cardiovascular diseases.

**Objective:**

We aimed to characterize all trials involving human subjects utilizing GT to treat noncongenital cardiovascular diseases.

**Methods:**

In March 2021, we searched for clinical trials on the ClinicalTrials.gov (CT), International Clinical Trials Registry Platform (ICTRP), and International Standard Randomised Controlled Trials Number (ISRCTN) databases. Two authors screened the titles and registry notes of all the searched studies. We collected details of the included studies regarding their design, location funding source, treated conditions, completion, publication statuses, and final outcomes.

**Results:**

We generated a total of 3508 records, and 50 unique clinical trials met our eligibility criteria. Of these, 20 (40%) concerned peripheral artery disease, and 18 (36%) concerned coronary artery disease. Most studies were randomized (34/50, 68%) and were performed in multiple locations (30/50, 60%), and around half of the trials compared GT with a placebo (27/50, 54%), while one in four were single-arm (14/50, 28%), and the rest concerned dose-finding (22%). More than half of the trials (29/50, 58%) were funded by industry. Of the 50 clinical trials, 28 (56%) published their results by the data collection date (March 2021), and 22 of 31 (71%) were slated to be completed before 2021. Overall, 12 of 28 (42.9%) clinical trials showed favorable outcomes of the intervention.

**Conclusions:**

Among noncongenital cardiovascular diseases, GTs are mostly investigated in peripheral artery disease and coronary artery disease. Many clinical trials on GT use in noncongenital cardiovascular diseases did not disclose their results. Regardless of the trial phase, less than half of published studies on GT in noncongenital cardiovascular diseases showed promising results.

## Introduction

Cardiovascular diseases remain the leading cause of morbidity and mortality worldwide, despite developments in treatment and diagnostics. In 2005, they were responsible for 29.7% of global deaths and for 32.1% in 2015 [1]. Therefore, there is a need for novel strategies to prevent and treat cardiovascular diseases and mitigate their consequences. Gene therapies (GTs) are methods of gene modification or expression to achieve specific cellular effects [2]. This novel class of therapeutics may improve the prevention and treatment of many diseases, including cardiovascular diseases. One of the first approved GTs was alipogene tiparvovec (Glybera; AMT-011, AAV1-LPLS447X), aimed at adults with familial lipoprotein lipase deficiency [3,4]. The potential of GT in noncongenital cardiovascular diseases was studied from the early 21st century, but its efficacy was limited [5,6]. In the past 15 years, several promising phase II randomized controlled trials (RCTs) of GT in heart failure (HF) [7,8] and peripheral artery disease (PAD) [9-11] were published. However, none of those GTs are currently approved (eg, NV1FGF for PAD failed to reduce major amputation or death in a phase III RCT [12]). In 2011, GT (Neovasculgen; cambiogenplasmid, PL-VEGFR165) for PAD was approved in Russia [13]. A postapproval observation of PL-VEGFR165 (Neovasculgen; cambiogenplasmid) suggested persistence of the therapeutic effect for individuals with PAD [13]. However, in the United States and Europe, there is currently no GT approved for noncongenital cardiovascular diseases. RCTs are considered the most reliable method for assessing the efficacy of therapeutics [14,15] and should also be used in gene therapy research. However, interventional trials may have many limitations in terms of design, sample selection, and end points. Moreover, it has been observed that many scientific groups do not publish the results of their clinical trials [16-20]. Nonpublication wastes scientists’ funds and efforts to generate data. Clinical trials are expensive, and participants are exposed to adverse events; thus, they should be precisely designed and their results published. If the results of previous trials are unavailable, other scientists have limited opportunities to assess the benefits and risks of similar interventions. Performing a study with a similar intervention may expose participants to unnecessary risk. Taken together, the publication of clinical trial results, even if negative, is not only an important part of the scientific process but also an ethical imperative. Gupta et al [20] analyzed obstetrical and gynecological RCTs registered on ClinicalTrials.gov between 2009 and 2013 and found that less than two-thirds of the trials were completed and only one-third published. The last known review of previous and ongoing clinical trials of GT in PAD, coronary artery diseases (CAD), and HF was performed in 2017 [6]. Since then, no study has described all clinical trials using GTs among noncongenital cardiovascular diseases.

In this cross-sectional study, we aimed to characterize all trials involving human participants utilizing GT for the treatment of noncongenital cardiovascular diseases.

## Methods

### Ethics Approval

This is a cross-sectional analysis of clinical trial registries and processes found in publicly available data and does not involve human or animal subjects. Therefore, the project did not require Ethical Committee approval. The analysis does not violate the terms of service of the registries used.

### Data Collection

We generated a list of clinical trials involving those registered at ClinicalTrials.gov (CT), the International Clinical Trials Registry Platform (ICTRP), and the International Standard Randomised Controlled Trial Number (ISRCTN) databases. We included all studies up to March 15, 2021, which was the date of the data collection. We included only trials that met the following eligibility criteria: (1) those that involved human participants, (2) where at least one of the study arms received gene therapy, and (3) where the study aimed to treat noncongenital cardiovascular disease according to the World Health Organization (WHO) definition [21].

In the CT database, we used the available filters as follows: other terms: “gene therapy,” study type: “interventional (clinical trial),” and conditions by category: “heart and blood diseases.” In other databases, we typed the phrase “gene therapy” into the search engine. We removed all “noninterventional” studies from .csv file generated from the ICTRP search engine. Furthermore, duplicated registered clinical trials were excluded.

Two authors (WP and ST) independently screened the titles and registry notes of all the generated studies. A third researcher (author MK) resolved any disputes or discrepancies after the initial classification. Additionally, we removed duplicated registered clinical trials.

### Data Processing and Statistical Analysis

We read the clinical trial design (study record details) published on the registry and publication if available. We then extracted self-reported study characteristics (eg, study design, trial location, funding source, treated condition, intervention, comparator, age of participants, sample size, initiation date, completion date, completion status, publication status, and outcome measures). In the case of incomplete reporting in some study records, we checked publications for missing information. Trials that were not categorized as single or multicenter were assigned to appropriate groups based on location. If the study claimed to be completed but no publication was linked in the registry, we searched for the registry number, study title, or keywords in PubMed and Google Scholar. We analyzed probable matches for trial design, location, sample size, therapy name, and date of publishing results. We considered those studies with posted results in the registry record or publication in a peer-reviewed journal as published. For all papers with published results, we searched for favorable outcomes. Favorable outcomes were defined as reaching either of the following primary aims: (1) optimal dose was established in early clinical trials, (2) GT showed acceptable safety profile, or (3) GT was better than the comparator in the primary aim or reached the primary aim in single-arm studies. We did not perform a risk of bias assessment on individual studies. In addition, we performed descriptive statistics. The primary outcome was the proportion of completed studies with favorable outcomes. The secondary outcomes included descriptive statistics of the studies’ analyzed features. Furthermore, we compared features of clinical trials that either published or did not publish their results. We included only trials that aimed to be completed before the day of data collection, which was March 15, 2021. We used the chi-square test for categorical variables and the Mann-Whitney U test for numerical variables.

## Results

**Figure 1:**
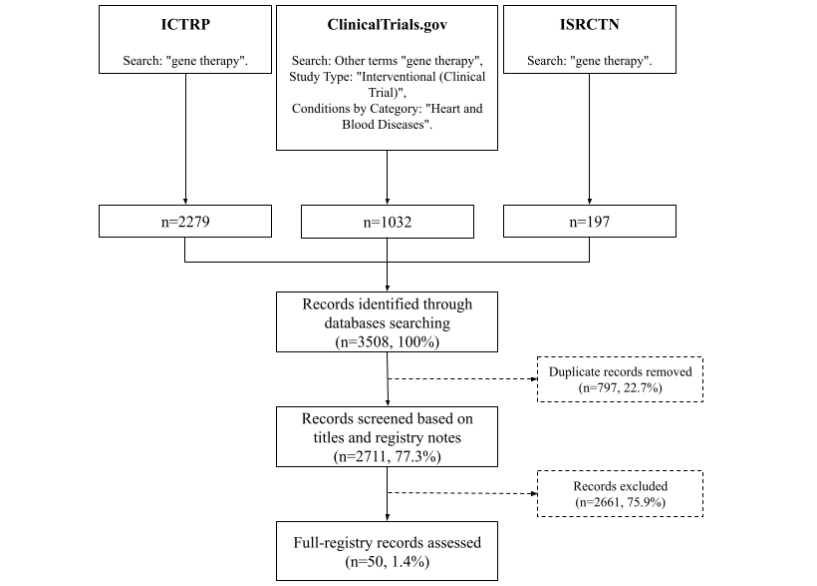
Data collection flowchart. ICTRP: International Clinical Trials Registry Platform, ISRCTN: International Standard Randomised Controlled Trials Number.

We generated a total of 3508 records through database searching: 1032 records from CT, 2279 from ICTRP, and 197 from ISRCTN (Figure 1). We screened 2711 records based on titles and registry notes. A total of 50 trials with 4436 total participants ultimately met our eligibility criteria. In Table 1, we present the characteristics of the included studies. We distinguished four groups depending on the treated disease: (1) PAD, (2) CAD, (3) HF, and (4) Other (pulmonary hypertension, ischemic cardiomyopathy, and secondary Raynaud's phenomenon). The predominant group was PAD, represented by 20 out of 50 (40%) studies, followed by CAD, with 18 (36%) studies. Most studies (34/50, 68%) were randomized and were performed in multiple places (30/50, 60%), and around half of the trials compared GT with the placebo (27/50, 54%), while one in four were single-arm (14/50, 28%), and the rest concerned dose finding (22%). More than half of the trials (29/50, 58%) were funded by industry. To date, 28 (56%) of the 50 clinical trials published their results (NCT02016755, JPRN-jRCTs053180162, JPRN-UMIN000014918) [7-13,22-40]. Of these, 12 (42.9%) revealed a favorable outcome of the interventions.

We present the results for all analyzed trials in Multimedia Appendices 1-4. One study (NCT00438867; AWARE study; phase III) only recruited females, while the rest included both sexes. All the studies involved only adults. Most studies had wide age ranges, but 2 studies (NCT00956332, NCT00566657) included individuals at least 50 years old. Most of the studies (34/50, 68%) were initiated within the last 10 years (2012-2021). A total of 31 trials were slated to finish before the date of data collection (March 2021). Of these, 22 (71%) were published. Three publications came from ongoing trials with completion dates after 2021 and three from projects with unknown completion dates. The most prevalent vectors were plasmids (25/50, 50%) and adenoviruses (18/50, 36%). GTs were mostly delivered via intramuscular injection (19/50, 38%), intramyocardial injection (17/50, 34%), and intracoronary infusion (11/50, 22%). Eighteen of 50 (36%) GTs transferred vascular endothelial growth factor (VEGF) genes. We did not find information about deaths related to the used GT. All collected variables are presented jointly in Multimedia Appendix 5.

We performed a comparison between published and unpublished clinical trials that should be completed before March 2021 (Table 2). We identified that the published clinical trials were initiated and completed in later years than those that were unpublished. We did not detect any significant differences in the other analyzed variables.

**Table 1:** Characteristics of included studies on gene therapies in noncongenital cardiovascular studies.

		Total	Peripheral arterial disease	Coronary artery disease	Heart failure	Other
Studies, n (%)		50 (100)	20 (40)	18 (36)	10 (20)	2 (5)
Total participants, n (%)		4436 (100)	2136 (48)	2034 (46)	216 (5)	50 (1)
**Study status, n (%)**						
	Completed	24 (48)	14 (70)	6 (33)	4 (40)	0 (0)
	Not completed	18 (36)	5 (25)	7 (39)	5 (50)	1 (50)
	Terminated	2 (6)	0 (0)	1 (6)	1 (10)	0 (0)
	NA^a^	6 (12)	1 (5)	4 (22)	0 (0)	1 (50)
**Funding, n (%)**						
	Industry	29 (58)	15 (75)	8 (44)	6 (60)	0 (0)
	Other	18 (36)	5 (25)	10 (56)	1 (10)	2 (100)
	Both	3 (6)	0 (0)	0 (0)	3 (30)	0 (0)
**Randomization, n (%)**						
	Yes	34 (68)	13 (65)	13 (72)	7 (70)	1 (50)
	No	13 (26)	5 (25)	5 (28)	3 (30)	0 (0)
	NA	3 (6)	2 (10)	0 (0)	0 (0)	1 (50)
**Center, n (%)**						
	Single center	15 (30)	6 (30)	5 (28)	2 (20)	2 (100)
	Multicenter	30 (60)	12 (60)	12 (67)	6 (60)	0 (0)
	NA	5 (10)	2 (10)	1 (6)	2 (20)	0 (0)
**Published, n (%)**						
	Yes	28 (56)	13 (65)	8 (44)	6 (60)	1 (50)
	No	22 (44)	7 (35)	10 (56)	4 (40)	1 (50)
**Comparator, n (%)**						
	Placebo	27 (54)	11 (55)	10 (56)	6 (60)	0 (0)
	Dose finding	11 (22)	5 (25)	4 (22)	2 (20)	0 (0)
	None	14 (28)	5 (25)	4 (22)	3 (30)	2 (100)
**Study phase, n (%)**						
	I	9 (18)	4 (20)	2 (11)	3 (30)	0 (0)
	I/II	11 (22)	2 (10)	4 (22)	4 (40)	1 (50)
	II	19 (38)	8 (40)	8 (44)	2 (20)	1 (50)
	II/III	2 (5)	1 (5)	1 (5.6)	0 (0)	0 (0)
	III	6 (12)	3 (15)	2 (11)	1 (10)	0 (0)
	NA	3 (6)	2 (10)	1 (6)	0 (0)	0 (0)
**Continent, n (%)**						
	North America	17 (34)	5 (25)	6 (33)	6 (60)	0 (0)
	Asia	13 (26)	9 (45)	4 (22)	0 (0)	0 (0)
	Europe	12 (24)	1 (5)	7 (39)	2 (20)	1 (50)
	South America	2 (4)	1 (5)	0 (0)	0 (0)	1 (50)
	Intercontinental	4 (8)	3 (15)	1 (6)	0 (0)	0 (0)
	NA	2 (4)	0 (0)	0 (0)	2 (20)	0 (0)
**Favorable outcomes (n=28), n (%)**						
	Yes	12 (43)	5 (36)	3 (38)	4 (67)	0 (0)
	No	16 (57)	9 (64)	5 (62)	2 (33)	0 (0)

^a^NA: nonavailable

**Table 2:** Comparison between published and unpublished clinical trials on gene therapy in noncongenital cardiovascular diseases completed before data collection (March 2021).

Features	Published (n=22)	Unpublished (n=9)	*P* value
Conditions, n (%)	Peripheral artery disease, 11 (50) Coronary artery disease, 5 (23) Heart failure, 5 (23) Other, 1 (5)	Peripheral artery disease, 4 (44) Coronary artery disease, 3 (33) Heart failure, 1 (11) Other, 1 (11)	.58
Number of participants, median (IQR)	49.5 (11-100)	12.0 (10-52)	.29
Phases, n (%)	I, 4(18) I/II, 3 (14) II, 10 (46) II/III, 2 (9) III, 1 (5)	I, 4 (44) I/II, 2 (22) II, 2 (22) II/III, 0 (0) III, 0 (0)	.53
Funded by, n (%)	Industry, 12 (55) Other, 7 (32) Both, 3 (14)	Industry, 6 (67) Other, 3 (33) Both, 0 (0)	.50
Randomized	14 (70)	5 (56)	>.99
Start date, median (IQR)	2007 (2004-2012)	2015 (2010-2018)	.02
Completion date, median (IQR)	2012 (2009-2015)	2016.0 (2013-2020)	.03
Continent, n (%)	Asia, 4 (18) Europe, 7 (32) North America, 8 (36) South America, 1 (5) Intercontinental, 2 (9)	Asia, 2 (22) Europe, 1 (11) North America, 4 (44) South America, 1 (11) Intercontinental, 1 (11)	.80
Single center study, n (%)	11 (50)	3 (33.3)	.58
Vector, n (%)	Plasmid, 14 (64) Virus, 4 (18)	Plasmid, 7 (78) Virus, 2 (22)	>.99

## Discussion

### Principal Findings

In this paper, we characterized all clinical trials on GTs for noncongenital cardiovascular diseases. The trials concerned mostly PAD and CAD, and most had positive traits in terms of good design, including randomization, multicenter design, and placebo as the comparator. Over half of all included clinical trials disclosed their results.

We found that most trials searched for efficient GTs for the atherosclerotic cardiovascular diseases PAD and CAD. GT in PAD was used to stimulate angiogenesis to heal ulcers or increase pain-free distance [6]. The pathophysiology of PAD and CAD results from atherosclerotic cardiovascular disease. Progressive occlusion of small vessels in the heart or limbs causes a decrease of tissue perfusion and consequently cell hypoxia and necrosis. Angiogenesis stimulated by VEGFs offers a potential approach for improving ischemic tissue function by inducing blood vessel growth to restore perfusion and regeneration. Similarly, the stimulation of angiogenesis CAD can improve myocardial perfusion and tolerability of physical activity. However, GT has limited success against these diseases [6]. The most recent meta-analysis from 2013 on GT in PAD did not find clear benefits from the treatment [41]. However, the high number of clinical trials on GT atherosclerotic cardiovascular diseases provides hope that effective GTs will be developed.

The primary cause of HF is a progressive loss of contractile function, which can be caused by both ischemic and nonischemic factors. Understanding how these factors affect heart function is key to developing an effective and targeted treatment. Regardless of its etiology, there is decreased sarcoplasmic reticulum Ca2+-ATPase (SERCA2a) activity in heart failure [42]. Abnormalities in the relaxation and contraction of cardiomyocytes are associated with calcium levels and reduced SERCA2a activity, and they can be treated by increasing the SERCA2a activity. In the database, we found six studies that used GT with adenoviral vector gene transfer to improve SERCA2a function. A leading cause in HF pathophysiology is an ischemic factor—CAD. The progression of atherosclerosis causes a decrease in the blood supply to cardiomyocytes, tissue damage, and decreased cardiac contractility. Potential treatment methods are VEGFs, which enhance angiogenesis in the ischemic heart [5]. These studies were conducted without favorable clinical outcomes. Another potential GT to treat ischemic cardiomyocytes is stem cell-derived factor 1 (SDF-1). SDF-1 recruits bone marrow-derived stem cells to the site of myocardial injury in a failing heart, where it induces tissue repair [43]. Desensitization of β-adrenergic receptors is another cause of HF, which could be targeted at the molecular level. Downregulation of receptors causes a weaker contraction response and lower levels of cAMP inside cardiomyocytes. This was treated by administering an adenoviral vector, which improved cAMP expression and activation of adenyl cyclase type 6 [44], showing promising results. GT in HF relies mostly on optimizing myocardial contraction and excitation processes and reducing myocardial wall remodeling [5,6,45]. To date, there are no registered GTs on HF, but this area of research is intensively developed, with 4 of 6 published clinical trials on GT in HF revealing favorable outcomes. Therefore, we can expect that future trials will bolster the possibilities of GT in cardiology.

Furthermore, we analyzed the publication rate of the included clinical trials. Approximately one in four completed clinical trials in urology reported results [46]. Liu et al [47] found that only 34% of oncology interventional trials registered on ClinicalTrials.gov published their results. A similar proportion of published clinical trials were noted for obstetrical and gynecological RCTs [20]. A higher publication rate was observed in orthopedic trauma trials (43.2%) [48], National Institutes of Health–funded trials (46%) [18], and RCTs involving patients with rare diseases (48.3%) [49]. However, Bourgeois et al [50] found that up to 66.3% of clinical trials conducted between 2000 and 2006 on anticholesteremics, antidepressants, antipsychotics, proton-pump inhibitors, and vasodilators were published. Moreover, the majority (71%) of large RCTs were published [16]. Considering publication rates from the aforementioned trials, 71% (22/31) of the disclosed results of clinical trials on GT in noncongenital cardiovascular diseases were very high. We speculate that this may be the result of: (1) a high priority given to trials on GT, (2) pressure from sponsors, and (3) the high citation potential of GT trials. However, ~30% of clinical trials that were planned to be completed before 2021 did not disclose their results. We found that unpublished trials were initiated and completed later than those published, which is similar to the previous observations [49]. Moreover, we found that many variables were not described in <10% of the clinical trial records.

In addition, we found that most clinical trials revealed positive traits of good design: 68% (34/50) were randomized, 60% (30/50) were multicenter studies, and 54% (27/50) used a placebo as a comparator. Interestingly, the reported outcomes were mostly negative (16/28, 57%). In the study by Bourgeois et al [50], drug trials funded by industry showed positive outcomes in 85.4% of publications, nonprofit or nonfederal organizations in 71.9%, and government-funded in 50%. However, a higher number of negative trials on GT in noncongenital cardiovascular disease may be caused by the relatively high rate of trials disclosing results.

### Future Directions

Further studies may deploy contemporary technologies, including artificial intelligence (AI), to simplify medical data processing. Human-like tasks can now be performed by machines (eg, image analysis, computer-aided diagnosis, patterns, and cost of health care studies), augmenting clinicians' and researchers' work. The power of AI may discover unmeasured confounders and associations impacting publication rates. Therefore, such analyses may help reduce health care costs while improving the overall morbidity and mortality associated with noncongenital cardiovascular diseases [51]. For that to be accomplished, there is a need for a large-scale study based on a database dedicated specifically for RCTs on novel gene therapies instead of several noncompatible databases containing incomplete information about RCT characteristics [52]. It should contain complete data to enable research on factors affecting the publishing rate, outcomes of treatments, quality, and costs of RCTs.

### Limitations

We acknowledge several limitations of the study. First, many of the analyzed clinical trial records had missing information. Second, the number of trials that should have been completed before the data collection date was low. For this reason, we could not perform a multivariate logistic regression analysis to indicate independent factors associated with results disclosure. Finally, we did not contact investigators of the trials to verify the status of the projects and the reason for termination or nonpublication of the trial.

### Conclusions

Among noncongenital cardiovascular diseases, GTs are mostly investigated in PAD and CAD. Many clinical trials on GT use in noncongenital cardiovascular diseases did not disclose their results. Regardless of the trial phase, less than half of published studies on GT in noncongenital cardiovascular diseases showed promising results.

## Appendix

Multimedia Appendix 1 Characteristics of included studies on gene therapies in peripheral artery disease.

Multimedia Appendix 2 Characteristics of included studies on gene therapies in coronary artery disease.

Multimedia Appendix 3 Characteristics of included studies on gene therapies in heart failure.

Multimedia Appendix 4 Characteristics of included studies on gene therapies in ischemic cardiomyopathy and secondary Raynaud's phenomenon.

Multimedia Appendix 5 Detailed characteristics of included studies on gene therapies in noncongenital cardiovascular studies.

## Abbreviations

AI: artificial intelligence
CAD: coronary artery disease
CT: ClinicalTrials.gov
GT: gene therapy
HF: heart failure
ICTRP: International Clinical Trials Registry Platform
ISRCTN: International Standard Randomised Controlled Trials Number
PAD: peripheral artery disease
RCT: randomized controlled trial
SDF-1: stem cell-derived factor 1
SERCA2a: sarcoplasmic reticulum Ca2+-ATPase
VEGF: vascular endothelial growth factor
WHO: World Health Organization

## Data Availability

The data sets used and analyzed in this study are available from the corresponding author on reasonable request.

## References

[1] GBD 2015 Mortality and Causes of Death Collaborators (2016). Global, regional, and national life expectancy, all-cause mortality, and cause-specific mortality for 249 causes of death, 1980-2015: a systematic analysis for the Global Burden of Disease Study 2015. Lancet.

[2] FDA; 2018. What is Gene Therapy? Internet.

[3] Stroes ES, Nierman MC, Meulenberg JJ, Franssen R, Twisk J, Henny CP, Maas MM, Zwinderman AH, Ross C, Aronica E, High KA, Levi MM, Hayden MR, Kastelein JJ, Kuivenhoven JA (2008). Intramuscular administration of AAV1-lipoprotein lipase lowers triglycerides in lipoprotein lipase–deficient patients. ATVB.

[4] Scott LJ (2015). Alipogene tiparvovec: a review of its use in adults with familial lipoprotein lipase deficiency. Drugs.

[5] Hulot J, Ishikawa K, Hajjar RJ (2016). Gene therapy for the treatment of heart failure: promise postponed. Eur Heart J.

[6] Ylä-Herttuala S, Bridges C, Katz MG, Korpisalo P (2017). Angiogenic gene therapy in cardiovascular diseases: dream or vision?. Eur Heart J.

[7] Zsebo K, Yaroshinsky A, Rudy JJ, Wagner K, Greenberg B, Jessup M, Hajjar RJ (2014). Long-term effects of AAV1/SERCA2a gene transfer in patients with severe heart failure. Circ Res.

[8] Hammond HK, Penny WF, Traverse JH, Henry TD, Watkins MW, Yancy CW, Sweis RN, Adler ED, Patel AN, Murray DR, Ross RS, Bhargava V, Maisel A, Barnard DD, Lai NC, Dalton ND, Lee ML, Narayan SM, Blanchard DG, Gao MH (2016). Intracoronary gene transfer of adenylyl cyclase 6 in patients with heart failure. JAMA Cardiol.

[9] Gu Y, Cui S, Wang Q, Liu C, Jin B, Guo W, Liu C, Chu T, Shu C, Zhang F, Han C, Liu Y (2019). A randomized, double-blind, placebo-controlled phase II study of hepatocyte growth factor in the treatment of critical limb ischemia. Molecular Therapy.

[10] Grossman PM, Mendelsohn F, Henry TD, Hermiller JB, Litt M, Saucedo JF, Weiss RJ, Kandzari DE, Kleiman N, Anderson RD, Gottlieb D, Karlsberg R, Snell J, Rocha-Singh K (2007). Results from a phase II multicenter, double-blind placebo-controlled study of Del-1 (VLTS-589) for intermittent claudication in subjects with peripheral arterial disease. American Heart Journal.

[11] Nikol S, Baumgartner I, Van Belle E, Diehm C, Visoná A, Capogrossi MC, Ferreira-Maldent N, Gallino A, Graham Wyatt M, Dinesh Wijesinghe L, Fusari M, Stephan D, Emmerich J, Pompilio G, Vermassen F, Pham E, Grek V, Coleman M, Meyer F (2008). Therapeutic angiogenesis with intramuscular NV1FGF improves amputation-free survival in patients with critical limb ischemia. Molecular Therapy.

[12] Belch J, Hiatt WR, Baumgartner I, Driver IV, Nikol S, Norgren L, Van Belle E, TAMARIS CommitteesInvestigators (2011). Effect of fibroblast growth factor NV1FGF on amputation and death: a randomised placebo-controlled trial of gene therapy in critical limb ischaemia. Lancet.

[13] Deev R, Plaksa I, Bozo I, Mzhavanadze N, Suchkov I, Chervyakov Y, Staroverov I, Kalinin R, Isaev A (2018). Results of 5-year follow-up study in patients with peripheral artery disease treated with PL-VEGF165 for intermittent claudication. Ther Adv Cardiovasc Dis.

[14] Hariton E, Locascio JJ (2018). Randomised controlled trials - the gold standard for effectiveness research: Study design: randomised controlled trials. BJOG.

[15] Torgerson C, Torgerson D, Taylor C, Newcomer Kathryn E., Hatry Harry P., Wholey Joseph S. (2015). Randomized Controlled Trials. Handbook of Practical Program Evaluation Internet Hoboken, NJ.

[16] Jones CW, Handler L, Crowell KE, Keil LG, Weaver MA, Platts-Mills TF (2013). Non-publication of large randomized clinical trials: cross sectional analysis. BMJ.

[17] Shepshelovich D, Goldvaser H, Wang L, Abdul Razak AR (2018). Comparison of published and unpublished phase I clinical cancer trials: an analysis of the CliniclTrials.gov database. Invest New Drugs.

[18] Ross JS, Tse T, Zarin DA, Xu H, Zhou L, Krumholz HM (2012). Publication of NIH funded trials registered in ClinicalTrials.gov: cross sectional analysis. BMJ.

[19] Manzoli L, Flacco ME, D'Addario M, Capasso L, De Vito C, Marzuillo C, Villari P, Ioannidis JPA (2014). Non-publication and delayed publication of randomized trials on vaccines: survey. BMJ.

[20] Gupta M, Petsalis M, Powers K, Chen H, Chauhan SP, Wagner S (2020). Randomized clinical trials in obstetrics-gynecology registered at ClinicalTrials.gov: characteristics and factors associated with publication. Eur J Obstet Gynecol Reprod Biol.

[21] (2021). Cardiovascular diseases (CVDs). Cardiovascular diseases (CVDs) Internet.

[22] Hartikainen J, Hassinen I, Hedman A, Kivelä Antti, Saraste A, Knuuti J, Husso M, Mussalo H, Hedman M, Rissanen T, Toivanen P, Heikura T, Witztum J, Tsimikas S, Ylä-Herttuala Seppo (2017). Adenoviral intramyocardial VEGF-DΔNΔC gene transfer increases myocardial perfusion reserve in refractory angina patients: a phase I/IIa study with 1-year follow-up. Eur Heart J.

[23] Ripa RS (2006). Intramyocardial injection of vascular endothelial growth factor-A165 plasmid followed by granulocyte-colony stimulating factor to induce angiogenesis in patients with severe chronic ischaemic heart disease. European Heart Journal.

[24] Hulot J, Salem J, Redheuil A, Collet J, Varnous S, Jourdain P, Logeart D, Gandjbakhch E, Bernard C, Hatem SN, Isnard R, Cluzel P, Le Feuvre C, Leprince P, Hammoudi N, Lemoine FM, Klatzmann D, Vicaut E, Komajda M, Montalescot G, Lompré A, Hajjar RJ (2017). Effect of intracoronary administration of AAV1/SERCA2a on ventricular remodelling in patients with advanced systolic heart failure: results from the AGENT-HF randomized phase 2 trial. Eur J Heart Fail.

[25] Penny WF, Henry TD, Watkins MW, Patel AN, Hammond HK (2018). Design of a Phase 3 trial of intracoronary administration of human adenovirus 5 encoding human adenylyl cyclase type 6 (RT-100) gene transfer in patients with heart failure with reduced left ventricular ejection fraction: The FLOURISH Clinical Trial. American Heart Journal.

[26] Lyon AR, Babalis D, Morley-Smith AC, Hedger M, Suarez Barrientos A, Foldes G, Couch LS, Chowdhury RA, Tzortzis KN, Peters NS, Rog-Zielinska EA, Yang H, Welch S, Bowles CT, Rahman Haley S, Bell AR, Rice A, Sasikaran T, Johnson NA, Falaschetti E, Parameshwar J, Lewis C, Tsui S, Simon A, Pepper J, Rudy JJ, Zsebo KM, Macleod KT, Terracciano CM, Hajjar RJ, Banner N, Harding SE (2020). Investigation of the safety and feasibility of AAV1/SERCA2a gene transfer in patients with chronic heart failure supported with a left ventricular assist device – the SERCA-LVAD TRIAL. Gene Ther.

[27] Stewart DJ, Hilton JD, Arnold JMO, Gregoire J, Rivard A, Archer SL, Charbonneau F, Cohen E, Curtis M, Buller CE, Mendelsohn FO, Dib N, Page P, Ducas J, Plante S, Sullivan J, Macko J, Rasmussen C, Kessler PD, Rasmussen HS (2006). Angiogenic gene therapy in patients with nonrevascularizable ischemic heart disease: a phase 2 randomized, controlled trial of AdVEGF(121) (AdVEGF121) versus maximum medical treatment. Gene Ther.

[28] Creager MA, Olin JW, Belch JJ, Moneta GL, Henry TD, Rajagopalan S, Annex BH, Hiatt WR (2011). Effect of hypoxia-inducible factor-1α gene therapy on walking performance in patients with intermittent claudication. Circulation.

[29] Kim JS, Hwang HY, Cho KR, Park E, Lee W, Paeng JC, Lee DS, Kim H, Sohn D, Kim K (2012). Intramyocardial transfer of hepatocyte growth factor as an adjunct to CABG: phase I clinical study. Gene Ther.

[30] Henry TD, Hirsch AT, Goldman J, Wang YL, Lips DL, McMillan WD, Duval S, Biggs TA, Keo HH (2011). Safety of a non-viral plasmid-encoding dual isoforms of hepatocyte growth factor in critical limb ischemia patients: a phase I study. Gene Ther.

[31] Kukuła K, Urbanowicz A, Kłopotowski M, Dąbrowski M, Pręgowski J, Kądziela J, Chmielak Z, Witkowski A, Rużyłło W (2019). Long-term follow-up and safety assessment of angiogenic gene therapy trial VIF-CAD: Transcatheter intramyocardial administration of a bicistronic plasmid expressing VEGF-A165/bFGF cDNA for the treatment of refractory coronary artery disease. Am Heart J.

[32] Stewart DJ, Kutryk MJ, Fitchett D, Freeman M, Camack N, Su Y, Siega AD, Bilodeau L, Burton JR, Proulx G, Radhakrishnan S (2009). VEGF gene therapy fails to improve perfusion of ischemic myocardium in patients with advanced coronary disease: results of the NORTHERN trial. Molecular Therapy.

[33] Giusti II, Rodrigues CG, Salles FB, Sant'Anna RT, Eibel B, Han SW, Ludwig E, Grossman G, Prates PRL, Sant'Anna JRM, Filho GFT, Markoski MM, Nesralla IA, Nardi NB, Kalil RAK (2013). High doses of vascular endothelial growth factor 165 safely, but transiently, improve myocardial perfusion in no-option ischemic disease. Hum Gene Ther Methods.

[34] Kastrup J, Jørgensen E, Fuchs S, Nikol S, Bøtker HE, Gyöngyösi M, Glogar D, Kornowski R (2011). A randomised, double-blind, placebo-controlled, multicentre study of the safety and efficacy of BIOBYPASS (AdGVVEGF121.10NH) gene therapy in patients with refractory advanced coronary artery disease: the NOVA trial. EuroIntervention.

[35] Niebuhr A, Henry T, Goldman J, Baumgartner I, van Belle E, Gerss J, Hirsch AT, Nikol S (2011). Long-term safety of intramuscular gene transfer of non-viral FGF1 for peripheral artery disease. Gene Ther.

[36] Van Belle E, Nikol S, Norgren L, Baumgartner I, Driver V, Hiatt WR, Belch J (2011). Insights on the role of diabetes and geographic variation in patients with critical limb ischaemia. Eur J Vasc Endovasc Surg.

[37] Penn MS, Mendelsohn FO, Schaer GL, Sherman W, Farr M, Pastore J, Rouy D, Clemens R, Aras R, Losordo DW (2013). An open-label dose escalation study to evaluate the safety of administration of nonviral stromal cell-derived factor-1 plasmid to treat symptomatic ischemic heart failure. Circ Res.

[38] Flugelman MY, Halak M, Yoffe B, Schneiderman J, Rubinstein C, Bloom A, Weinmann E, Goldin I, Ginzburg V, Mayzler O, Hoffman A, Koren B, Gershtein D, Inbar M, Hutoran M, Tsaba A (2017). Phase Ib safety, two-dose study of MultiGeneAngio in patients with chronic critical limb ischemia. Molecular Therapy.

[39] Meng H, Chen B, Tao Z, Xu Z, Wang L, Weizhu J, Hong Y, Liu X, Wang H, Wang L, Wu Z, Yang Z (2018). Safety and efficacy of adenovirus carrying hepatocyte growth factor gene by percutaneous endocardial injection for treating post-infarct heart failure: a phase IIa clinical trial. Curr Gene Ther.

[40] Yonemitsu Y, Matsumoto T, Itoh H, Okazaki J, Uchiyama M, Yoshida K, Onimaru M, Onohara T, Inoguchi H, Kyuragi R, Shimokawa M, Ban H, Tanaka M, Inoue M, Shu T, Hasegawa M, Nakanishi Y, Maehara Y (2013). DVC1-0101 to treat peripheral arterial disease: a Phase I/IIa open-label dose-escalation clinical trial. Mol Ther.

[41] Hammer A, Steiner S (2013). Gene therapy for therapeutic angiogenesis in peripheral arterial disease - a systematic review and meta-analysis of randomized, controlled trials. Vasa.

[42] Gwathmey JK, Copelas L, MacKinnon R, Schoen FJ, Feldman MD, Grossman W, Morgan JP (1987). Abnormal intracellular calcium handling in myocardium from patients with end-stage heart failure. Circ Res.

[43] Ghadge SK, Mühlstedt S, Ozcelik C, Bader M (2011). SDF-1α as a therapeutic stem cell homing factor in myocardial infarction. Pharmacol Ther.

[44] Lai NC, Roth DM, Gao MH, Tang T, Dalton N, Lai YY, Spellman M, Clopton P, Hammond HK (2004). Intracoronary adenovirus encoding adenylyl cyclase VI increases left ventricular function in heart failure. Circulation.

[45] Greenberg B (2015). Gene therapy for heart failure. Journal of Cardiology.

[46] Magnani CJ, Steinberg JR, Harmange CI, Zhang X, Driscoll C, Bell A, Larson J, You JG, Weeks BT, Hernandez-Boussard T, Turner BE, Brooks JD (2021). Clinical trial outcomes in urology: assessing early discontinuation, results reporting and publication in ClinicalTrials.Gov registrations 2007-2019. J Urol.

[47] Liu X, Zhang Y, Li W, Vokes E, Sun Y, Le Q, Ma J (2021). Evaluation of oncology trial results reporting over a 10-year period. JAMA Netw Open.

[48] Gandhi R, Jan M, Smith HN, Mahomed NN, Bhandari M (2011). Comparison of published orthopaedic trauma trials following registration in Clinicaltrials.gov. BMC Musculoskelet Disord.

[49] Rees CA, Pica N, Monuteaux MC, Bourgeois FT (2019). Noncompletion and nonpublication of trials studying rare diseases: A cross-sectional analysis. PLoS Med.

[50] Bourgeois FT, Murthy S, Mandl KD (2010). Outcome reporting among drug trials registered in ClinicalTrials.gov. Ann Intern Med.

[51] Amritphale A, Chatterjee R, Chatterjee S, Amritphale N, Rahnavard A, Awan GM, Omar B, Fonarow GC (2021). Predictors of 30-day unplanned readmission after carotid artery stenting using artificial intelligence. Adv Ther.

[52] Valentín LI, Márquez E (1986). Persistent interstitial pulmonary emphysema. Bol Asoc Med P R.

