# Supplementary material for: Characteristics and Outcomes of Clinical Trials on Gene Therapy in Noncongenital Cardiovascular Diseases: Cross-sectional Study of Three Clinical Trial Registries
Source: JMIR Form Res. 2022 Apr 21;6(4):e33893. doi: 10.2196/33893 (PMC9073605; doi:10.2196/33893)
Supplement: Multimedia Appendix 1 [file formative_v6i4e33893_app1.doc]

**Multimedia Appendix 1.** Characteristics of included studies on gene therapies in peripheral artery disease.

A)

| **ID** | **NCT00798005** | **JPRN-C000000404** | **NCT00696124** | **NCT01064440** | **NCT00956332** | **NCT02016755** |
| --- | --- | --- | --- | --- | --- | --- |
| **Completed** | YES | YES | YES | YES | NO | YES |
| **Phases** | 2 | 1/2 | 1 | 2 | 1/2 | 2 |
| **Age** | ≥45 | ≥40 | 20-90 | 18-90 | 50-90 | 40-90 |
| **Enrollment** | 71 | 12 | 15 | 52 | 28 | 10 |
| **Funded By** | industry | other | industry | industry | industry | industry |
| **Randomized** | YES | NO | NO | YES | YES | NA |
| **Start Date** | 2002 | 2006 | 2007 | 2010 | 2010 | 2013 |
| **Completion Date** | 2005 | NA | 2010 | 2013 | 2026 | 2018 |
| **Continent** | North America | Asia | North America | North America, Asia | Asia | North America |
| **Single center study** | YES | NA | YES | NO | NO | YES |
| **Primary aims** | Incidence of adverse events | Incidence of adverse events | Incidence of adverse events | Incidence of adverse events | Incidence of adverse events | Incidence of adverse events |
| **Therapy name** | XRP0038/NV1FGF | DVC-0101 | VM202 | VM202 | MultiGeneAngio | AMG0001 |
| **Vector** | plasmid | Sendai Virus | plasmid | plasmid | NA | plasmid |
| **Delivery method** | i.m. - lower limb | i.m. - lower limb | i.m. - lower limb | i.m. - lower limb | i.a. - lower limb | i.m. - lower limb |
| **Delivered gene** | FGF-1 | FGF-2 | HGF-X7 | HGF-X7 | Angiogenic genes | HGF |
| **Published** | YES | YES | YES | NO | YES | YES |
| **Favorable outcome** | NO | YES | NO | NA | YES | NO |
| **Comparator** | placebo | dose-escalation | dose finding | placebo | dose-escalation | None |
| **Death related to treatment** | 0 | 0 | 0 | NA | 0 | NA |

NA - nonavailable

B)

| **ID** | **NCT00117650** | **NCT03068585** | **JPRN-jRCTs053180162** | **NCT04275323** | **NCT04274049** | **NCT03668353** | **NCT04110964** |
| --- | --- | --- | --- | --- | --- | --- | --- |
| **Completed** | YES | YES | YES | NO | NO | NA | NO |
| **Phases** | 2 | 2/3 | NA | 3 | 3 | 1 | 1 |
| **Age** | 40-80 | 40-70 | 20-85 | 20-80 | 20-80 | 18-80 | >45 |
| **Enrollment** | 289 | 100 | 6 | 300 | 240 | 12 | 5 |
| **Funded By** | industry | other | industry | industry | industry | industry | industry |
| **Randomized** | YES | YES | NO | YES | YES | NO | NA |
| **Start Date** | 2005 | 2010 | 2014 | 2019 | 2019 | 2018 | 2019 |
| **Completion Date** | 2010 | 2011 | 2019 | 2022 | 2022 | 2020 | 2020 |
| **Continent** | North America, Europe | Europe | Asia | Asia | Asia | Asia | South America |
| **Single center study** | NO | NO | NA | NO | NO | YES | YES |
| **Primary aims** | Increase in pain-free walking distance | Increase in pain-free walking distance | Improvement of pain at rest and ischemic ulcer | Complete pain relief rate | Percentage of ulcer complete healing | Incidence of adverse events | Incidence of adverse events |
| **Therapy name** | Ad2/HIF-1α/VP16 | Neovasculgen | AMG0001 | NL003-CLI-III-1 | NL003 | Recombinant SeV-hFGF2/dF | AAV-hTERT |
| **Vector** | adenovirus | CMV | plasmid | plasmid | plasmid | Sendai Virus | adenovirus |
| **Delivery method** | i.m. - lower limb | i.m. - lower limb | i.m. - lower limb | i.m. - lower limb | i.m. - lower limb | i.m. - lower limb | i.v. |
| **Delivered gene** | HIF-1α | VEGF165 | HGF | HGF-X7 | HGF-X7 | FGF2 | hTERT |
| **Published** | YES | YES | YES | NO | NO | NO | NO |
| **Favorable outcome** | NO | NO | YES | NA | NA | NA | NA |
| **Comparator** | placebo | None | None | placebo | placebo | dose-escalation | None |
| **Death related to treatment** | 0 | 8, not classified if related | NA | NA | NA | NA | NA |

NA - nonavailable

C)

| **ID** | **NCT00368797** | **NCT00068133** | **NCT00566657** | **NCT01548378** | **NCT02276937** | **JPRN-UMIN000014918** | **NCT00080392** |
| --- | --- | --- | --- | --- | --- | --- | --- |
| **Completed** | YES | YES | YES | YES | NO | YES | YES |
| **Phases** | 2 | 2 | 3 | 2 | 2 | NA | 1 |
| **Age** | ≥40 | 40-80 | ≥50 | 30-80 | ≥30 | 20-85 | ≥21 |
| **Enrollment** | 125 | 100 | 525 | 200 | 30 | 6 | 10 |
| **Funded By** | industry | industry | industry | industry | other | other | other |
| **Randomized** | YES | YES | YES | YES | YES | NO | YES |
| **Start Date** | 2002 | 2003 | 2007 | 2012 | 2014 | 2014 | 2004 |
| **Completion Date** | 2005 | 2005 | 2012 | 2014 | 2022 | 2019 | 2011 |
| **Continent** | Europe | North America | Europe, North America, | Asia | Asia | Asia | North America |
| **Single center study** | NO | NO | NO | NO | NO | YES | NO |
| **Primary aims** | Percentage of ulcer complete healing | Increase in pain-free walking distance | Time to major amputation of the treated leg or death from any cause | The difference in ulcer area between baseline and the D180 | Increase in pain-free walking distance | Improvement of pain at rest and ischemic ulcer | NA |
| **Therapy name** | XRP0038 | VLTS-589 | XRP0038/NV1FGF | NL003 | DVC1-0101 | AMG0001 | EW-A-401 |
| **Vector** | plasmid | plasmid | plasmid | plasmid | Sendai Virus | plasmid | plasmid |
| **Delivery method** | i.m. - lower limb | i.m. - lower limb | i.m. - lower limb | i.m. - lower limb | i.m. - lower limb | i.m. - lower limb | i.m. - lower limb |
| **Delivered gene** | FGF-1 | angiomatrix protein Del-1 | FGF-1 | HGF-X7 | FGF-2 | HGF | VEGF-A |
| **Published** | YES | YES | YES | YES | NO | YES | NO |
| **Favorable outcome** | NO | NO | NO | NO | NA | YES | NA |
| **Comparator** | placebo | placebo | placebo | placebo | placebo, dose-escalation | None | placebo |
| **Death related to treatment** | NA | NA | NA | 0 | NA | 0 | NA |

NA - nonavailable
