# Supplementary material for: Characteristics and Outcomes of Clinical Trials on Gene Therapy in Noncongenital Cardiovascular Diseases: Cross-sectional Study of Three Clinical Trial Registries
Source: JMIR Form Res. 2022 Apr 21;6(4):e33893. doi: 10.2196/33893 (PMC9073605; doi:10.2196/33893)
Supplement: Multimedia Appendix 2 [file formative_v6i4e33893_app2.doc]

**Multimedia Appendix 2.** Characteristics of included studies on gene therapies in coronary artery disease.

A)

| **ID** | **NCT00143585** | **NCT00135850** | **NCT00090714** | **NCT00620217** | **NCT00215696** | **NCT00438867** | **NCT01550614** | **NCT02844283** |
| --- | --- | --- | --- | --- | --- | --- | --- | --- |
| **Completed** | NA | YES | NO | YES | YES | NA | NO Terminated | NA |
| **Phases** | 2/3 | 1/2 | 2 | 2 | 2 | 3 | 3 | 1/2 |
| **Age** | 18-75 | 20-80 | >18 | ≥18 | 18-80 | 18-75 | 18-75 | 30-80 |
| **Enrollment** | 120 | 48 | 404 | 52 | 129 | 300 | 11 | 100 |
| **Funded By** | other | other | industry | other | industry | industry | industry | other |
| **Randomized** | YES | NO | YES | YES | YES | YES | YES | YES |
| **Start Date** | 2002 | 2003 | 2004 | 2004 | 2005 | 2007 | 2012 | 2016 |
| **Completion Date** | 2007 | 2005 | NA | 2009 | NA | NA | NA | NA |
| **Continent** | North America | Europe | North America | Europe | Europe, Asia | North America | Asia | Asia |
| **Single center study** | NO | YES | NO | YES | NO | NO | NO | NA |
| **Primary aims** | Myocardial perfusion-stress/rest scores -SRS | Mobilization of stem cells | Determine the optimum dose | Change in myocardial perfusion at rest | Change on exercise tolerance test | Change in time to onset of ECG changes | Change in reversible perfusion defect size (RPDS) | Changes of cardiac left ventricular ejection fraction |
| **Therapy name** | VEGF165 | NA | pVGI.1(VEGF2) | VEGF-A165/bFGF | BIOBYPASS® | Ad5FGF-4 | Ad5FGF-4 | Ad-HGF |
| **Vector** | NA | plasmid | plasmid | Plasmid | Adenovirus | Adenovirus serotype 5 | Adenovirus serotype 6 | Adenovirus |
| **Delivery method** | Intramyocardial injection | intramyocardial injection | intramyocardial injection | Intramyocardial injection | Intramyocardial injection | Intracoronary infusion | Intracoronary infusion | Intracoronary infusion (infarct-related artery) |
| **Delivered gene** | VEGF165 | VEGF165 | pVGI.1(VEGF2) | basic FGF (bFGF) and VEGF type A | VEGF121 | FGF-4 | FGF-5 | HGF |
| **Published** | YES | YES | NO | YES | YES | NO | NO | YES |
| **Favorable outcome** | NO | NO | NA | NO | NO | NA | NA | YES |
| **Comparator** | placebo | None | dose finding | placebo | placebo | placebo | None | placebo |
| **Death related to treatment** | NA | 0 | NA | No full access | No full access | NA | NA | No full access |

NA - nonavailable

B)

| **ID** | **EUCTR2018-001494-24-DK** | **EUCTR2017-000789-31-FI** | **EUCTR2017-000789-31-DK** | **NCT03039751** | **ISRCTN87681696** | **NCT01422772** | **NCT01002430** | **NCT01002495** | **NCT03404024** | **NCT04125732** |
| --- | --- | --- | --- | --- | --- | --- | --- | --- | --- | --- |
| **Completed** | NA | NO | NO | NO | YES | YES | YES | NO | NO | NO |
| **Phases** | 2 | 2 | 2 | 2 | NA | 1 | 1 | 1/2 | 2 | 1/2 |
| **Age** | 30-85 | 30-85 | 30-85 | 30-85 | 18-75 | 19-75 | 39-80 | 21-75 | 19-75 | 18-80 |
| **Enrollment** | 30 | 180 | 180 | 180 | target 120 | 9 | 30 | 0 | 108 | 33 |
| **Funded By** | other | other | other | other | other | industry | other | industry | industry | industry |
| **Randomized** | NO | YES | YES | YES | YES | NO | YES | NO | YES | NO |
| **Start Date** | 2018 | 2019 | 2019 | 2019 | 2002 | 2007 | 2010 | 2015 | 2018 | 2020 |
| **Completion Date** | NA | 2023 | 2023 | 2023 | 2006 | 2014 | 2015 | 2016 | 2020 | 2022 |
| **Continent** | Europe | Europe | Europe | Europe | North America | Asia | Europe | North America | Asia | North America |
| **Single center study** | YES | NO | NO | NO | NO | YES | YES | NO | NO | NO |
| **Primary aims** | Change in myocardial angiogenesis | Improvement in walking distance | Improvement in walking distance | Improvement in 6-minute walking test | Incidence of adverse events | Incidence of adverse events | Incidence of adverse events | Incidence of adverse events | Incidence of adverse events | Incidence of adverse events |
| **Therapy name** | 68Ga-NODAGA-E[c(RGDyK)]2 | ReGenHeart | ReGenHeart | AdVEGF-D | NA | VM202RY | VEGF-D gene transfer | VM202 | VM202RY | XC001 |
| **Vector** | NA | Adenovirus | Adenovirus | Adenovirus | Plasmid | plasmid | AdVEGF-D adenovirus | plasmid | plasmid | AdVEGFXC1 adenovirus |
| **Delivery method** | NA | Intramyocardial injection | Intramyocardial injection | Intramyocardial injection | i.m. cardiac | intramyocardial injection | intramyocardial injection | intramyocardial injection | intramyocardial injection | intramyocardial injection |
| **Delivered gene** | VEGF-D | VEGF-D | VEGF-D | VEGF-D | VEGF 165 | HGF-X7 | VEGF-D | HGF-X7 | HGF-X7 | VEGF |
| **Published** | NO | NO | NO | NO | NO | YES | YES | NO | NO | YES |
| **Favorable outcome** | NA | NA | NA | NA | NA | YES | NO | NA | NA | YES |
| **Comparator** | None | placebo | placebo | placebo | placebo | dose finding | None | dose finding | placebo | dose finding |
| **Death related to treatment** | NA | NA | NA | NA | NA | 0 | 0 | NA | NA | 0 |

NA - nonavailable
