# Supplementary material for: Characteristics and Outcomes of Clinical Trials on Gene Therapy in Noncongenital Cardiovascular Diseases: Cross-sectional Study of Three Clinical Trial Registries
Source: JMIR Form Res. 2022 Apr 21;6(4):e33893. doi: 10.2196/33893 (PMC9073605; doi:10.2196/33893)
Supplement: Multimedia Appendix 3 [file formative_v6i4e33893_app3.doc]

**Multimedia Appendix 3.** Characteristics of included studies on gene therapies in heart failure.

| **ID** | **NCT00279539** | **NCT01082094** | **NCT02346422** | **NCT03409627** | **NCT03360448** | **NCT00454818** | **NCT00787059** | **NCT01966887** | **NCT00534703** | **NCT04703842** |
| --- | --- | --- | --- | --- | --- | --- | --- | --- | --- | --- |
| **Completed** | NO | YES | NO Terminated | YES | NO | YES | YES | NO | NO | NO |
| **Phases** | 1 | 1 | 1/2 | 1 | 3 | 1/2 | 1/2 | 2 | 2 | 1/2 |
| **Age** | >21 | ≥18 | 18-80 | ≥18 | 18-80 | 18-75 | 18-80 | 18-80 | 18-70 | 18-80 |
| **Enrollment** | 0 | 17 | 9 | 12 | 0 | 51 | 56 | 10 | 5 | 56 |
| **Funded By** | other | industry | industry | industry | industry | industry | both | both | both | industry |
| **Randomized** | NO | NO | YES | NO | YES | YES | YES | YES | YES | YES |
| **Start Date** | 2008 | 2010 | 2015 | 2018 | 2019 | 2007 | 2010 | 2013 | 2014 | 2021 |
| **Completion Date** | NA | 2012 | NA | 2020 | 2023 | 2012 | 2017 | 2016 | 2015 | 2028 |
| **Continent** | NA | North America | North America | North America | North America | North America | North America | Europe | Europe | NA |
| **Single center study** | NA | NO | NO | NO | NO | NO | NO | YES | YES | NA |
| **Primary aims** | Incidence of adverse events | Incidence of adverse events | Incidence of adverse events | Incidence of adverse events | Incidence of adverse events | Change in 6-minute Walk Test From Baseline to Month 6 | Exercise treadmill time | Left ventricular end-systolic volume | NA | NA |
| **Therapy name** | NA | ACRX-100/ JVS-100 | MYDICAR® | INXN-4001 | Ad5.hAC7 | MYDICAR® | Ad5.hAC6 | MYDICAR® | SERCA | MYDICAR® |
| **Vector** | plasmid | Plasmid | Adeno-associated viral vector | Plasmid | Ad5.hAC6 adenovirus | Adeno-associated viral vector | Ad5.hAC6 adenovirus | AAV1-CMV-SERCA2a adenovirus | AAV1/SERCA2a adenovirus | adenovirus |
| **Delivery method** | intramyocardial injection | Intramyocardial injection | Intracoronary infusion | Intracoronary infusion | intracoronary infusion | Intracoronary infusion | intracoronary infusion | intracoronary infusion | intracoronary infusion | intracoronary infusion |
| **Delivered gene** | VEGF165 | SDF-1 | SERCA2a | S100A1, SDF-1a, VEGF-A165. | adenylyl cyclase type 6 (AC6) | SERCA2a | adenylyl cyclase type 6 (AC6) | SERCA2a | SERCA2a | AAV1/SERCA2a |
| **Published** | NO | YES | NO | NO | YES | YES | YES | YES | YES | NO |
| **Favorable outcome** | NA | YES | NA | NA | YES | YES | YES | NO | NO | NA |
| **Comparator** | None | dose-escalation | placebo | None | placebo | placebo, dose-escalation | placebo | None | placebo | placebo |
| **Death related to treatment** | NA | 0 | NA | NA | 0 | 0 | 0 | NA | 0 | NA |

NA - nonavailable
