# Supplementary material for: Characteristics and Outcomes of Clinical Trials on Gene Therapy in Noncongenital Cardiovascular Diseases: Cross-sectional Study of Three Clinical Trial Registries
Source: JMIR Form Res. 2022 Apr 21;6(4):e33893. doi: 10.2196/33893 (PMC9073605; doi:10.2196/33893)
Supplement: Multimedia Appendix 4 [file formative_v6i4e33893_app4.doc]

**Multimedia Appendix 4.** Characteristics of included studies on gene therapies in ischemic cardiomyopathy, and secondary Raynaud's phenomenon.

| **ID** | **NCT00744315** | **NCT02356809** |
| --- | --- | --- |
| **Disease** | Ischemic cardiomyopathy | Secondary Raynaud's Phenomenon |
| **Completed** | NA | NO |
| **Phases** | 2 | 1/2 |
| **Age** | ≤75 | 18-65 |
| **Enrollment** | 20 | 30 |
| **Funded By** | other | other |
| **Randomized** | NA | YES |
| **Start Date** | 2007 | 2015 |
| **Completion Date** | 2009 | 2015 |
| **Continent** | South America | Europe |
| **Single center study** | YES | YES |
| **Primary aims** | SPECT, treadmill tests, Minnesota quality of life questionnaire (QOL), NYHA, CCS scale | Frequency of new digital ulcers |
| **Therapy name** | hVEGF165 | Neovasculgen |
| **Vector** | Plasmid | plasmid |
| **Delivery method** | Intramyocardial injection | Intramuscular injection - hand |
| **Delivered gene** | VEGF165 | VEGF165 |
| **Published** | YES | NO |
| **Favorable outcome** | YES | NA |
| **Comparator** | None | None |
| **Death related to treatment** | 0 | NA |

NA - nonavailable
